# Supplementary figures and images for: A Comprehensive Identification and Expression Analysis of VQ Motif-Containing Proteins in Sugarcane (Saccharum spontaneum L.) under Phytohormone Treatment and Cold Stress
Source: Int J Mol Sci. 2022 Jun 6;23(11):6334. doi: 10.3390/ijms23116334 (PMC9181594; doi:10.3390/ijms23116334)

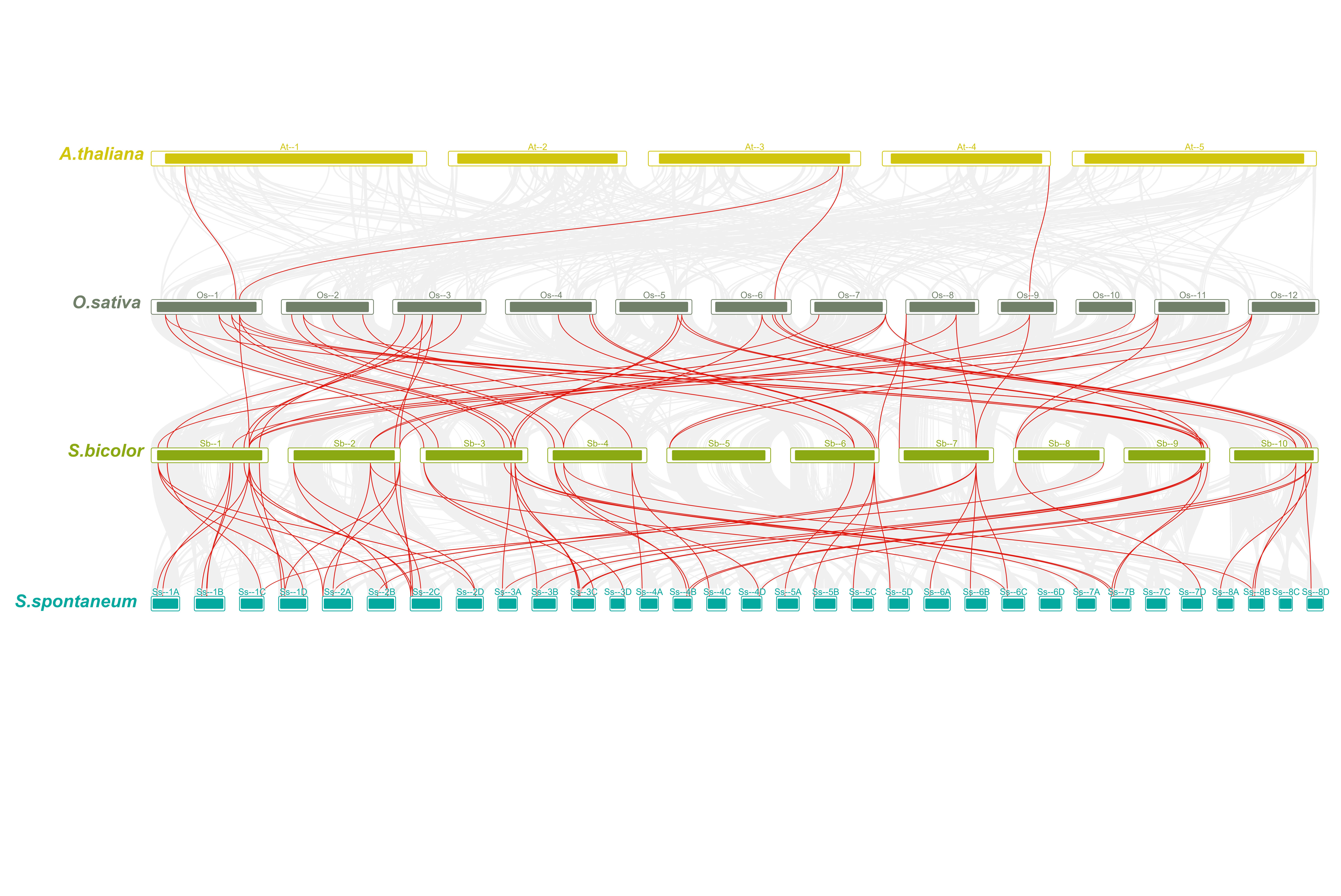

Supplement: Supplementary file 1 [file ijms-23-06334-s001.zip › Fig.S1 Synteny analysis .jpg]
